# Detrimental consequences after intimal disruption of subclavian artery during transcathether aortic valve implantation

**DOI:** 10.1186/s13019-023-02131-6

**Published:** 2023-01-12

**Authors:** Oliver T. Reuthebuch, Ion Vasiloi, Thomas Nestelberger, Thomas Wolff, Friedrich S. Eckstein

**Affiliations:** 1 Department of Cardiac Surgery, University Hospital Basel, Spitalstrasse 21, 4031 Basel, Switzerland; 2 Department of Cardiology, University Hospital Basel, Basel, Switzerland; 3 Department of Vascular Surgery, University Hospital Basel, Basel, Switzerland

**Keywords:** Aortic valve stenosis, Transcathater aortic valve implantation, Subclavian artery, Intimal disruption

## Abstract

**Background:**

TAVI via the left subclavian artery is considered a bail-out strategy in cases where a transfemoral approach is not feasible. However, since this route is only scarcely used, major complications can arise. We describe such an adverse course and present our proceeding.

**Case presentation:**

A 65-year-old man with severe aortic valve stenosis (AS) was referred for transcatheter aortic valve implantation (TAVI) via left subclavian artery. After uneventful deployment of the TAVI prosthesis, consequent valve assessment with transeosophageal echocardiography and angiography showed a highly mobile and tubular structure shifting within the valve. We went for a surgical extraction via sternotomy on cardiopulmonary bypass (CPB). A 6 cm longish intimal cylinder was hassle-free extracted. 4 days postoperatively the left sided radial pulse was missing. In a subsequent computed tomography angiography (CTA) scan a proximal dissection as well as an intimal flap, causing a subtotal stenosis of the left subclavian artery, was detected. Consecutively the intimal cylinder was removed using a Fogarty-balloon. Pre-discharge control revealed recurrence of peripheral radial pulse and an unimpeded function of the TAVI prosthesis. The patient presented no sequela at discharge.

**Conclusion:**

Though TAVI is a well-advanced technique complications are not completely avertable. It is thus advisable to have patients discussed in the heart team encompassing all potentially involved specialties.

**Supplementary Information:**

The online version contains supplementary material available at 10.1186/s13019-023-02131-6.

## Background

Transcatheter aortic valve implantation (TAVI) has become the preferred treatment option for patients with symptomatic severe aortic stenosis (AS) who are inoperable or at high risk for surgical aortic valve replacement [1]. Femoral access is by far the most frequent route for TAVI. Nevertheless, in cases of severe calcification, smallness or tortuosity of the femoral and/or iliac vessels alternative routes can be chosen [2]. Transsubclavian, direct aortic or transapical access present the most frequent option for TAVI implantation [3]. Despite the technical and processual evolution of TAVI techniques, the implantation can result in challenging conditions potentially requiring cardiac surgery support [4].

This report describes a unique case with intimal disruption of the subclavian artery with consequent displacement of the dissected cylinder into the TAVI prosthesis, surgical extirpation and further dissection and occlusion of the subclavian artery with catheter-based removal.

## Case presentation

A 65-year-old man with severe, symptomatic AS was referred to our heart team. The preoperative echocardiography showed a severely degenerated bicuspid AS with a mean gradient of 41 mmHg (LVEF 47%). With an estimated mortality of 10.12% (Euroscore II) based on various severe co-morbidities, the heart-team recommended an interventional aortic valve approach. Because of furthermore severely calcified and stenotic iliac arteries, an approach via left subclavian artery was chosen using a self-expandable valve (Evolut pro+; Medtronic, Minneapolis, Minnesotta, USA).

**Fig. 1:**
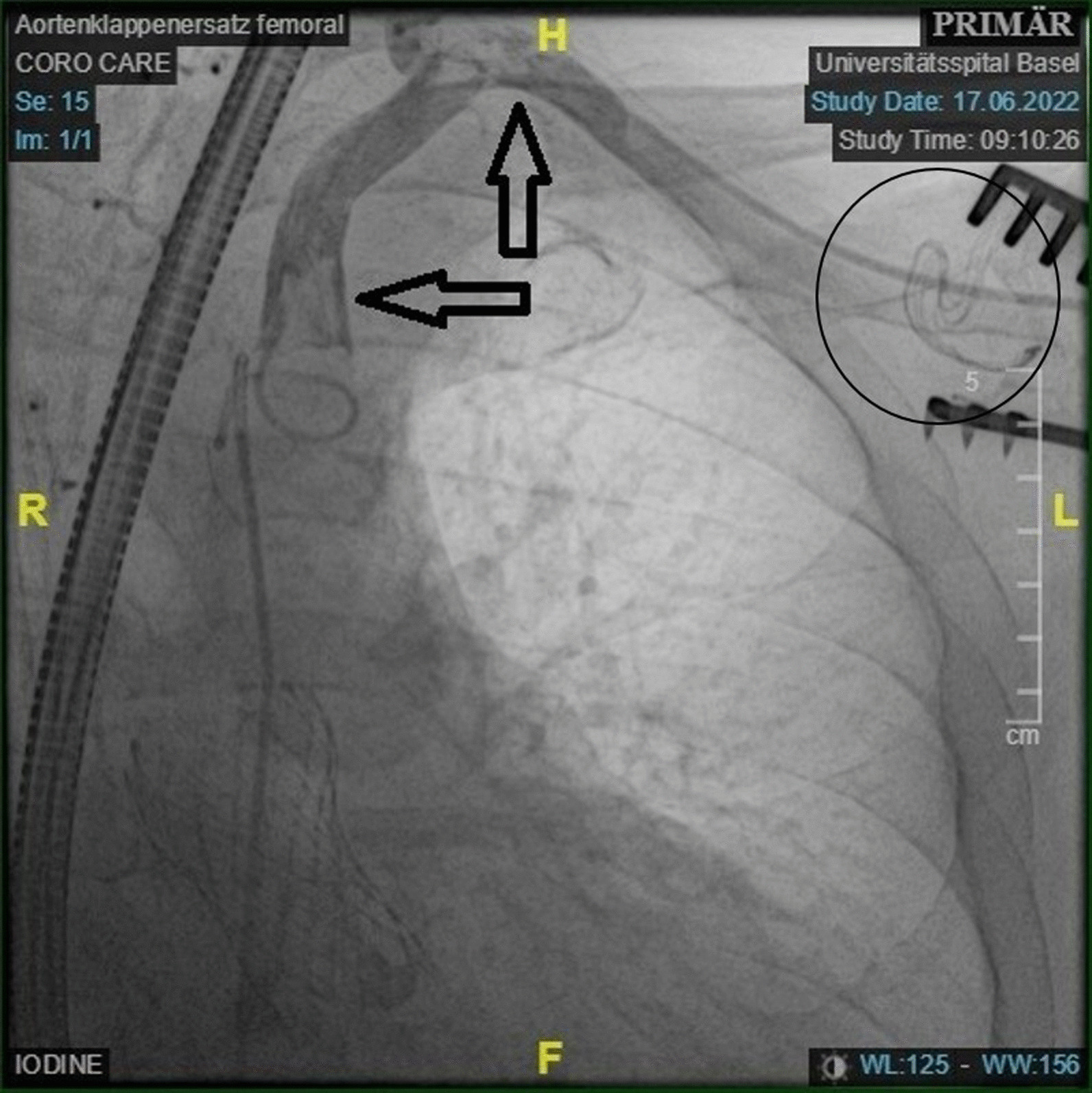
Intraoperative angiogram, showing the intimal tears in the subclavian artery (arrow), as well as the non-dissected clamp-site of the subclavian artery (circle)

**Fig. 2:**
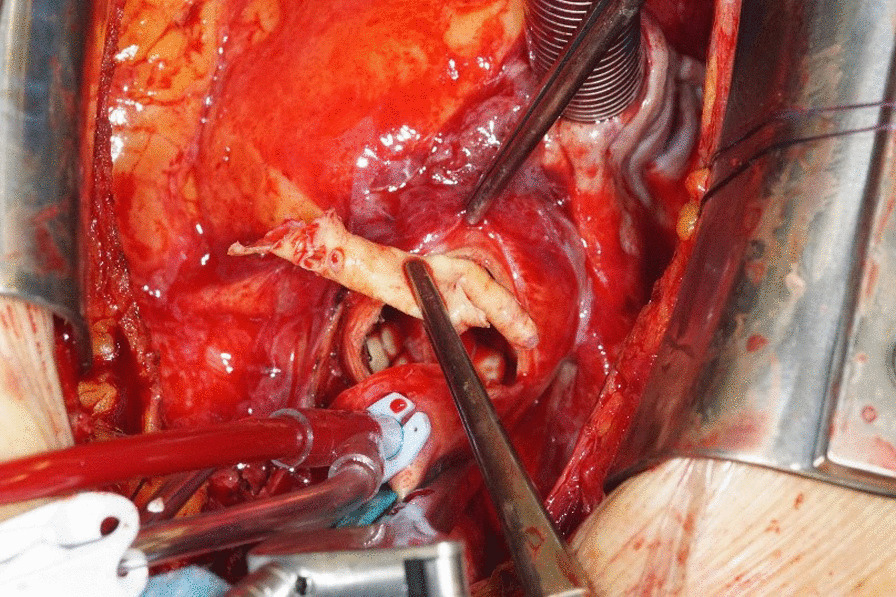
Intraoperative extraction of the intimal cylinder via the aortotomy under extracorporeal circulation

The procedure was performed in a hybrid operating room under general anesthesia. A transesophageal echocardiography (TEE) probe was inserted for periprocedural valve assessment. Via an incision in the infraclavicular fossa the left subclavian artery (8 mm in diameter) was exposed and an 8 mm Dacron tube was grafted for vascular access. The delivery sheath was introduced into the Dacron tube without passing the artery. By advancing the TAVI prosthesis under fluoroscopy within the subclavian artery an impediment at the level of the vertebral artery was sensed. The prosthesis was retracted and the location radiographically inspected. There was no severe kinking or harm to the vessel detected. Hence, the valve was reinserted and under slight resistance positioned in the annulus. After uneventful deployment of the prosthesis, function was assessed with TEE. It showed a 6 cm floating cylindrical structure in the course of the TAVI-prosthesis (Additional file 1). Suspecting some intravascular damage, a control angiogram was performed, displaying a filling defect of the subclavian artery (Fig. 1). We suspected an intimal tear caused by the valve insertion with subsequent dislocation and fixation in the struts of the TAVI-prosthesis. Since this highly mobile structure was considered to be potentially embolic, we proceeded to surgical extraction via sternotomy. Under CPB a median sternotomy was performed and routine cannulation for cardiopulmonary bypass was initiated. After aortic cross-clamping and cardioplegic arrest, aortotomy was performed. The intraoperative inspection revealed an intimal cylinder of the left subclavian artery nailed by the valve stent into the annulus, subsequently being completely removed (Fig. 2) leaving the correctly implanted TAVI in situ. Postoperatively, valve function (no regurgitation and a mean gradient of 2 mmHg) and blood circulation of the left arm were uncompromised.

**Fig. 3:**
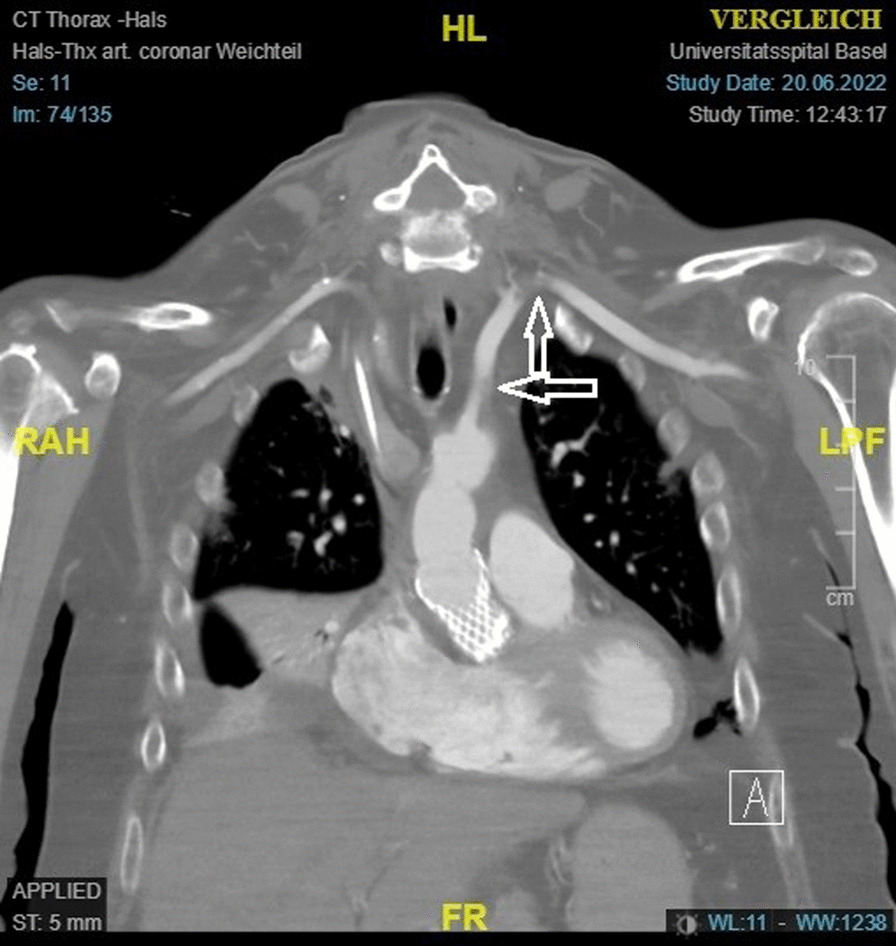
Postoperative CTA scan showing a dissection flap in the proximal left subclavian artery and contrast loss just distal to the junction of the left vertebral artery

**Fig. 4:**
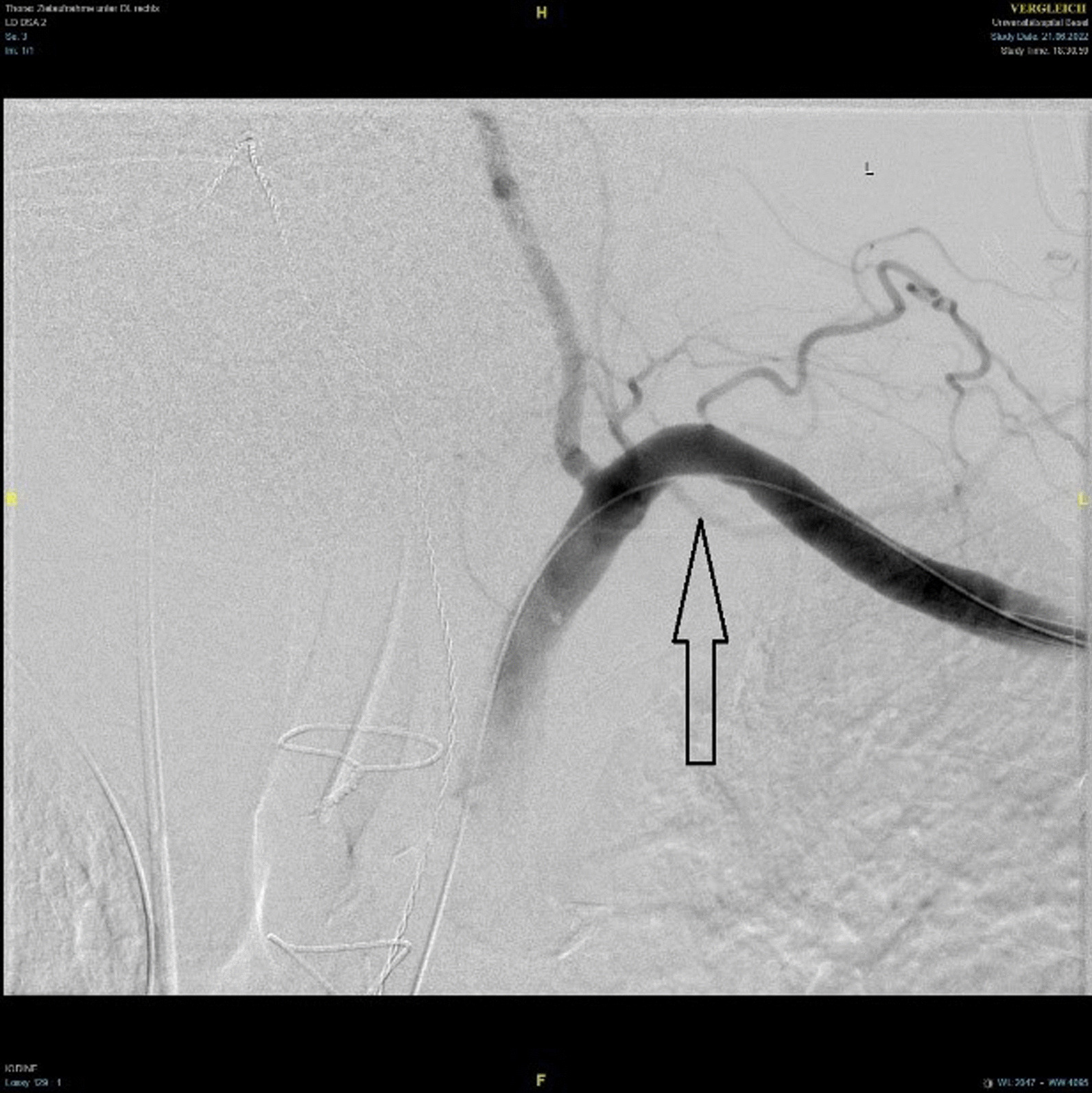
Digital subtracted angiography (DSA) showing restoration of flow in the subclavian artery after catheter-based extraction of the intimal flap

Yet, on postoperative day 4 absence of pulsation of the left-sided radial artery occurred. The CTA scan showed a dissection flap in the proximal left subclavian artery and distal contrast loss (Fig. 3). Catheter-based vascular intervention with Fogarty balloon-removal of the dissected intimal flap was performed. Postoperative control revealed return of peripheral pulse on the radial artery (Fig. 4). Further postoperative course was uneventful. The patient presented no new cardiac, vascular or neurologic sequelae at discharge.

## Discussion and conclusion

To our knowledge, this is the first such case released in literature. Due to an adverse femoral axis, we have chosen the subclavian approach for TAVI. In our institute, the subclavian access as described is the preferred bail-out for hostile femoral/iliac vessels with 33 performed within the last 5 years. Trans-subclavian access presents the third most used technique for TAVI implantation in Switzerland with only a minor difference between transapical approach (2%) and subclavian approach (1.1%) [5]. The implantation led to an intimal tearing of the left subclavian artery during insertion of the TAVI prosthesis, with subsequent dislocation into the TAVI prosthesis. Based on CT scan and angiography, there was at no time peri- or post-interventionally evidence for dissection at the level of clamp site of the subclavian artery. Since the patient was intubated, the diagnosis was made coincidentally by means of echocardiographic assessment. Angiographically no irregularities were encountered. Conversion to sternotomy and surgical extraction of the intimal plug was considered indispensible, since implantation of a second valve or removal with a snare were supposed to be too perilous due to embolization. Despite meticulous patient selection, preoperative screening and CTA based planning complications, requiring conversion to sternotomy or even vascular surgical interventions after TAVI are not completely avertable [6]. Therefore, the importance of interdisciplinary collaboration from the heart-team to the operation has to be repeatedly emphasized. Although intra-procedural complications with TAVI are scarce, implantation should be performed in tertiary hospital with onsite cardiac surgery and preferably in a hybrid operating room [4]. Since TAVI is an emerging valve therapy, its possible complications are still not fully elucidated.

## Supplementary Information

**Additional file 1:** **Video 1.** Floating intimal flap in the TAVI-valve.

## Data Availability

All data generated or analysed during this study are included in this published article.

## Abbreviations

AS: Aortic stenosis
CPB: Cardio pulmonary bypass
CTA: Computed tomography angiography
LVEF: Left ventricular ejection fraction
TAVI: Transcatheter aortic valve implantation
TEE: Trans esophageal echocardiography
